# IL-27-Dependent *Lag3* Regulates CD4^+^ Foxp3^+^ Regulatory T Cells to Alleviate Airway Inflammation in Allergic Asthma

**DOI:** 10.3390/ijms27146260

**Published:** 2026-07-14

**Authors:** Miaojuan Zhu, Rongyao Feng, Nishan Deng, Yifei Chen, Jiong Yang, Hanxiang Nie

**Affiliations:** 1 Department of Respiratory and Critical Care Medicine, Renmin Hospital of Wuhan University, Wuhan 430060, China; zhumiaoj@whu.edu.cn (M.Z.); nishandeng@whu.edu.cn (N.D.); 2 Department of Respiratory and Critical Care Medicine, Zhongnan Hospital of Wuhan University, Wuhan 430071, China; fengry1998@foxmail.com (R.F.); chenyf@whu.edu.cn (Y.C.)

**Keywords:** allergic asthma, IL-27, regulatory T cells, *Lag3*, airway inflammation

## Abstract

Allergic asthma is associated with a reduction in the number of regulatory T cells (Tregs). Although interleukin-27 (IL-27) has been shown to modulate Tregs potentially through the *Lymphocyte-activation gene 3* (*Lag3*) pathway, the underlying mechanism remains incompletely defined. Objective: This study sought to determine whether IL-27 ameliorates airway inflammation in asthma by modulating Tregs in a *Lag3*-dependent manner. Acute asthma was induced in *wild-type* (*WT*) and *Lag3* knockout (*Lag3*^−/−^) mice through sensitization and challenge with house dust mite (HDM). A treatment group received intranasal recombinant IL-27 prior to challenges. In *WT* mice, IL-27 administration significantly attenuated airway inflammation, goblet cell hyperplasia, and total cell counts in bronchoalveolar lavage fluid (BALF), along with reduced levels of Th2 cytokines (IL-4, IL-5). It also upregulated T-bet (Th1) mRNA expression, downregulated *GATA-3* (Th2) and *RORγt* (Th17) levels, and increased the proportions of CD4^+^ Foxp3^+^ Tregs, CTLA4^+^ Tregs, and *Lag3*^+^ Tregs in lung tissue. Conversely, in *Lag3*^−/−^ mice, the protective effects of IL-27 were completely abrogated, with no observed increases in Treg populations or suppression of Th2/Th17 immune responses. The anti-asthmatic effect of exogenous IL-27 is associated with increased Treg frequency and upregulation of inhibitory markers, with Lag3 serving as a pivotal target on Tregs.

## 1. Introduction

Bronchial asthma is a heterogeneous disease involving a diverse range of immune cells and cytokines. Following antigen recognition by antigen-presenting cells, effector Th2 cells produce interleukin (IL)-4, IL-5, and IL-13, which are key mediators of disease pathogenesis [1]. Foxp3^+^ regulatory T cells (Tregs) represent a critical T cell subset with specialized functions in maintaining immune self-tolerance and homeostasis [2]. In severe asthma, the number of Foxp3^+^ Tregs is significantly reduced compared to healthy controls, indicating an impairment in both Treg homeostasis and function during the development of allergic asthma [3]. Thus, strategies aimed at restoring Treg homeostasis or enhancing their regulatory function warrant further investigation.

IL-27, a member of the IL-12 cytokine family, consists of p28 and Ebi3 subunits [4]. Studies have shown that IL-27 expression is negatively correlated with Th2 cytokine levels in patients with allergic asthma [5]. Furthermore, intranasal administration of IL-27 in an allergic asthma model was found to increase the frequency of Foxp3^+^ Tregs and Foxp3 gene expression in cervical lymph nodes [6]. However, the mechanism by which IL-27 modulates Treg function in house dust mite (HDM)-induced acute allergic asthma remains unclear.

*Lymphocyte activation gene 3* (*Lag3*) is an immune checkpoint receptor expressed by activated/exhausted CD4^+^ and CD8^+^ T cells, as well as by Tregs. Emerging evidence suggests that *Lag3* enhances the inhibitory function of Tregs [7]. IL-27 has been shown to induce *Lag3* expression on Tregs in models of allergic airway inflammation [8]. Nevertheless, the functional contribution of *Lag3* to IL-27-mediated regulation of Tregs in suppressing allergic asthma inflammation has not been fully elucidated.

In this study, we hypothesized that IL-27 ameliorates allergic airway inflammation by promoting Treg expansion and function in a *Lag3*-dependent manner. We used a house dust mite (HDM)-induced acute asthma model in wild-type and *Lag3* knockout mice, with or without intranasal recombinant IL-27 treatment. Our aim was to determine whether *Lag3* is required for IL-27 to enhance Treg numbers, restore Th1/Th2/Th17 balance, and alleviate airway inflammation. The results of this study may provide a rationale for developing novel therapeutic strategies for allergic asthma by targeting the IL-27/*Lag3* pathway in Tregs.

## 2. Results

### 2.1. Exogenous Recombinant IL-27 Alleviates Allergic Airway Inflammation in an Acute Experimental Asthma Model

**Figure 1:**
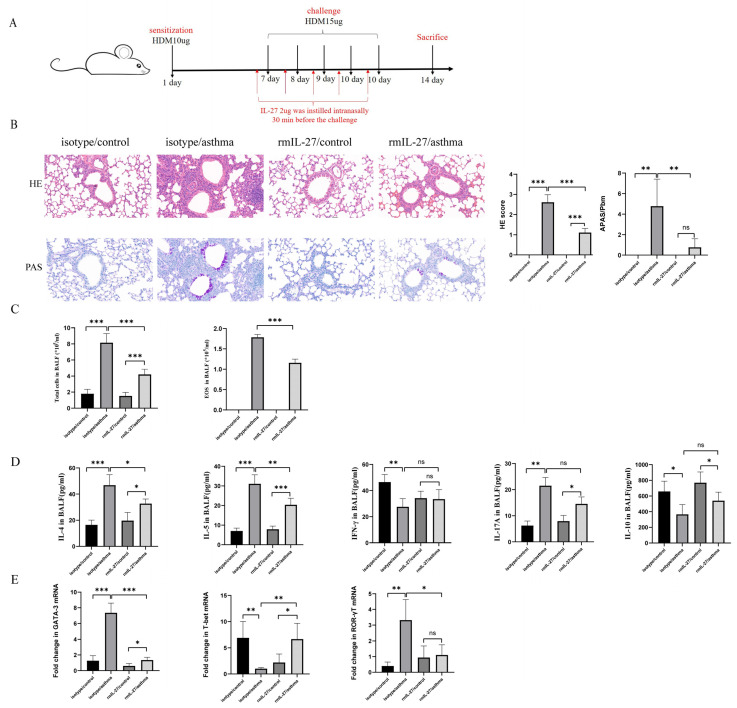
Intranasal administration of IL-27 ameliorates airway inflammation and modulates T-helper cell responses in a wild-type murine model of acute allergic asthma. (**A**) Experimental scheme for HDM-induced allergic asthma and IL-27 treatment protocol. (**B**) Representative micrographs (original magnification ×200) of H&E and PAS-stained lung sections from each group. (**C**) Total cell counts and eosinophil counts in bronchoalveolar lavage fluid (BALF) samples. (**D**) Levels of Th2 cytokines (IL-4, IL-5), a Th17-associated cytokine (IL-17A), a Th1-associated cytokine (IFN-γ), and IL-10 in BALF. (**E**) mRNA expression levels of Th2-associated transcription factor (GATA-3), Th1-associated transcription factor (T-bet), and Th17-associated transcription factor (ROR-γt). n = 5. * *p* < 0.05, ** *p* < 0.01, *** *p* < 0.001, ns, not significant.

A house dust mite (HDM)-induced mouse model of allergic asthma was established (Figure 1A). Histopathological evaluation of lung tissue via H&E and PAS staining revealed that IL-27-treated asthmatic mice exhibited reduced inflammatory cell infiltration, better-preserved alveolar architecture, and significantly less goblet cell hyperplasia compared to *wild-type* (*WT*) asthmatic mice (Figure 1B). Quantitative analysis showed that the H&E inflammation score was significantly lower in the IL-27-treated group (1.111 ± 0.202) than in the *WT* asthma group (2.611 ± 0.375) (*p* < 0.001). Similarly, the PAS-positive area per basement membrane length (APAS/Pbm) was markedly lower in IL-27-treated mice (0.760 ± 0.842) than in *WT* asthmatic controls (4.774 ± 2.639) (*p* < 0.01) (Figure 1B).

Analysis of bronchoalveolar lavage fluid (BALF) showed that the total cell count in IL-27-treated asthmatic mice was significantly reduced by approximately half compared to *WT* asthmatic mice (*p* < 0.001), accompanied by a marked decrease in eosinophil numbers (Figure 1C).

### 2.2. Exogenous Recombinant IL-27 Modulates the Balance of T Helper Cell Responses

Cytokine levels in BALF were measured by ELISA. IL-27-treated asthmatic mice showed significantly reduced levels of IL-4 and IL-5 (*p* < 0.05 or *p* < 0.01), whereas IL-17A and IFN-γ levels remained unchanged compared to *WT* asthmatic controls (*p* > 0.05). No significant difference was observed in IL-10 levels in either BALF or serum between the two groups (*p* > 0.05) (Figure 1D). 

qPCR analysis of lung tissue revealed that IL-27 treatment significantly upregulated T-bet mRNA expression (*p* < 0.01) and downregulated GATA-3 (*p* < 0.05) and RORγt (*p* < 0.001) expression in lung tissue (Figure 1E). Notably, the increase in T-bet mRNA did not lead to a corresponding elevation of IFN-γ protein, suggesting possible post-transcriptional regulation.

### 2.3. Exogenous Recombinant IL-27 Promotes an Increase in the Number of CD4^+^ Foxp3^+^ Treg Cells in Murine Lung Tissue

**Figure 2:**
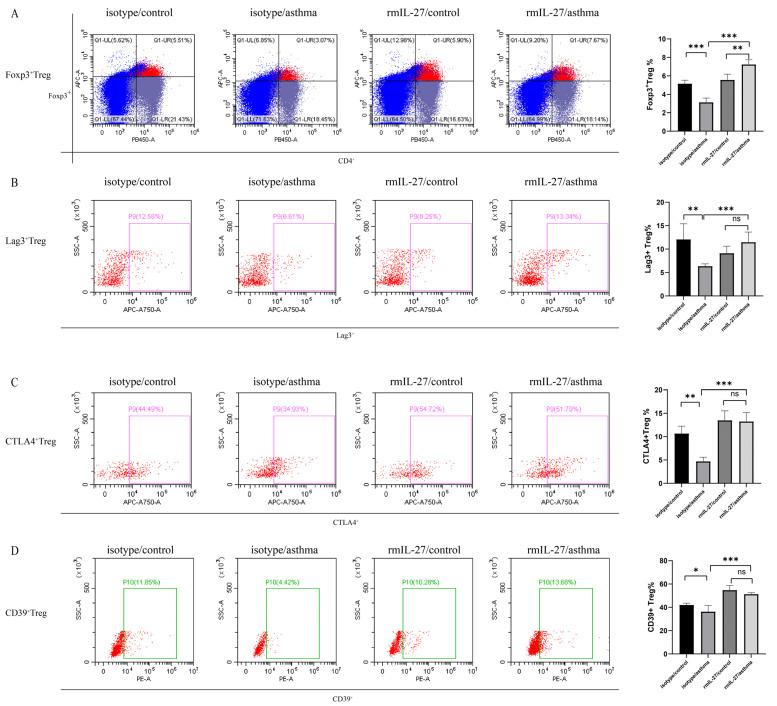
Intranasal IL-27 administration increases Treg frequency and upregulates inhibitory markers in a wild-type murine model of acute allergic asthma. (**A**) Representative flow cytometry plots and statistical analysis of Treg cells in mouse lung tissue. (**B**) Representative flow cytometry plots and proportional statistical analysis of *Lag3*^+^ Treg cells in mouse lung tissue. (**C**) Representative flow cytometry plots and statistical analysis of the proportion of CTLA4^+^ cells among Foxp3^+^ Treg cells in mouse lung tissue. (**D**) Representative flow cytometry plots and statistical analysis of the proportion of CD39^+^ cells among Foxp3^+^ Treg cells in mouse lung tissue. n = 5. * *p* < 0.05, ** *p* < 0.01, *** *p* < 0.001, ns, not significant.

Flow cytometric analysis demonstrated that the proportion of Tregs (CD4^+^ Foxp3^+^) among CD4^+^ T cells in lung tissue was similar between IL-27-treated and untreated *WT* normal mice (approximately 5.51% vs. 5.90%, *p* > 0.05). However, *WT* asthmatic mice showed a significant decrease in Treg frequency compared to normal controls (3.07% vs. 5.51%, *p* < 0.001). IL-27 treatment significantly increased the Treg ratio in asthmatic mice compared to untreated *WT* asthmatic controls (7.67% vs. 3.07%, *p* < 0.01), although it did not fully restore it to normal levels (Figure 2A).

### 2.4. Exogenous Recombinant IL-27 Upregulates the Expression of Lag3, CTLA4, and CD39 on CD4^+^ Foxp3^+^ Tregs

The expression of functional markers on Tregs was assessed by flow cytometry. No differences in the frequencies of CD39^+^ Tregs, CTLA4^+^ Tregs, or *Lag3*^+^ Tregs were observed between IL-27-treated and untreated normal mice. In *WT* asthmatic mice, the proportions of *Lag3*^+^ Tregs, CTLA4^+^ Tregs and CD39^+^ Tregs were significantly decreased (*p* < 0.05 or *p* < 0.01). IL-27 treatment significantly elevated the frequencies of *Lag3*^+^ Tregs, CTLA4^+^ Tregs and CD39^+^ Tregs in asthmatic mice (*p* < 0.001) (Figure 2B–D).

### 2.5. Lag3 Deficiency Abrogates the Protective Effect of IL-27 in Allergic Asthma

**Figure 3:**
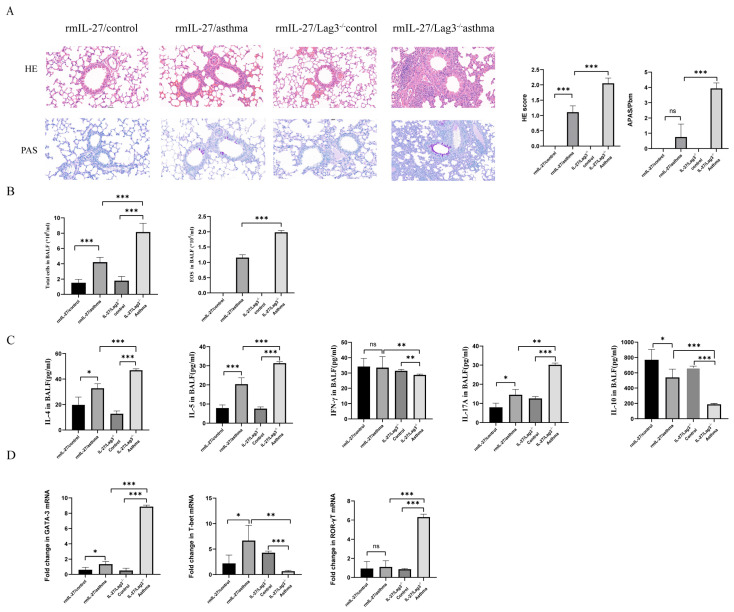
Intranasal IL-27 administration fails to alleviate airway inflammation or modulate T-helper cell responses in a *Lag3*-deficient murine model of acute allergic asthma. (**A**) Representative micrographs (original magnification ×200) of H&E and PAS-stained lung sections from each group. (**B**) Total cell counts and eosinophil counts in bronchoalveolar lavage fluid (BALF) samples. (**C**) Levels of Th2 cytokines (IL-4, IL-5), a Th17-associated cytokine (IL-17A), a Th1-associated cytokine (IFN-γ), and IL-10 in BALF. (**D**) mRNA expression levels of Th2-associated transcription factor (GATA-3), Th1-associated transcription factor (T-bet), and Th17-associated transcription factor (ROR-γt). n = 5. * *p* < 0.05, ** *p* < 0.01, *** *p* < 0.001, ns, not significant.

Histopathological analysis showed that IL-27-treated *Lag3*^−/−^ asthmatic mice exhibited more severe alveolar destruction, increased inflammatory cell infiltration with patchy aggregation, interstitial inflammation, and enhanced goblet cell hyperplasia compared to IL-27-treated *WT* asthmatic mice (Figure 3A). The H&E score was significantly higher in IL-27-treated *Lag3*^−/−^ asthmatic mice (2.306 ± 0.427) than in IL-27-treated *WT* asthmatic mice (1.111 ± 0.202, *p* < 0.001). The APAS/Pbm value in the *Lag3*^−/−^ group (3.941 ± 0.350) was significantly increased compared with that in the *WT* group (0.760 ± 0.842, *p* < 0.01) (Figure 3A).

BALF analysis revealed a significant increase in total cell count in IL-27-treated *Lag3*^−/−^ asthmatic mice compared to IL-27-treated *WT* asthmatic controls (*p* < 0.001), along with elevated eosinophil numbers (Figure 3B).

### 2.6. Lag3 Deficiency Impairs IL-27-Mediated Th Response Regulation

ELISA of BALF showed that IL-27-treated *Lag3*^−/−^ asthmatic mice had significantly higher levels of IL-4, IL-5, and IL-17A, and lower levels of IFN-γ and IL-10, compared to IL-27-treated *WT* asthmatic mice (*p* < 0.01) (Figure 3C). 

qPCR analysis indicated that T-bet expression was significantly downregulated (*p* < 0.05), while GATA-3 (*p* < 0.001) and RORγt (*p* < 0.01) expressions were significantly upregulated in IL-27-treated *Lag3*^−/−^ asthmatic mice compared to IL-27-treated *WT* asthmatic controls.

### 2.7. Lag3 Is Essential for Maintaining CD4^+^ Foxp3^+^ Treg Frequency

**Figure 4:**
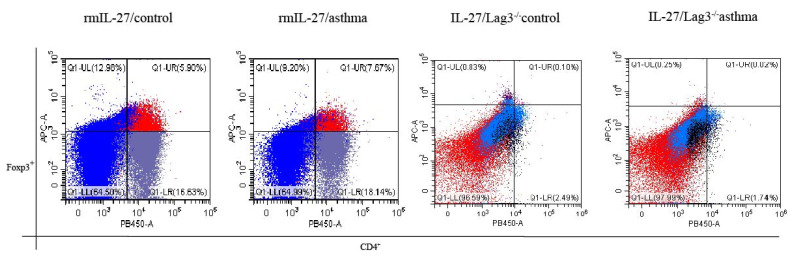
Intranasal IL-27 administration fails to increase Treg frequency in a *Lag3*-deficient murine model of acute allergic asthma. Representative flow cytometry plots showing Treg cells (CD4^+^FOXP3^+^) gated as Q1-UR (upper-right quadrant).

Flow cytometry revealed that the frequency of Tregs in lung tissue was significantly reduced in IL-27-treated *Lag3*^−/−^ mice compared to IL-27-treated *WT* mice (*p* < 0.001) and the asthma mice (*p* < 0.001). The severely low Treg numbers in *Lag3*-deficient mice precluded reliable assessment of CD39^+^ Treg and CTLA4^+^ Treg subpopulations (Figure 4).

## 3. Discussion

In this study, we systematically investigated the effect of IL-27 on Foxp3^+^ inducible Tregs using an HDM-induced acute allergic asthma model. Our key findings are: exogenous IL-27 significantly reduces airway inflammation, goblet cell hyperplasia, and alveolar damage; it downregulates Th2 cytokines (IL-4, IL-5) and transcription factors GATA-3 and RORγt, while upregulating T-bet; and it increases Treg frequency and upregulates inhibitory molecules CTLA4 and *Lag3* on Tregs. More importantly, in Lag3-deficient mice, all these protective effects are abolished, and Treg numbers cannot be restored, leading to uncontrolled Th2/Th17 responses. These results demonstrate for the first time in vivo that IL-27 relies on Lag3 to increase Treg numbers and upregulate their inhibitory markers, thereby controlling allergic airway inflammation.

Compared to ovalbumin (OVA), HDM induces a murine asthma model that more closely recapitulates human asthma pathology [9,10]. Our observation that IL-27 reduces inflammatory infiltration and goblet cell hyperplasia aligns with previous reports [11,12], supporting IL-27 as an endogenous negative regulator. Notably, IL-27 was administered 30 min before each challenge (preventive regimen), which differs from clinical therapeutic settings. Thus, future studies should evaluate IL-27 in established asthma models.

Asthma is characterized by Th1/Th2 imbalance. Our data confirmed that IL-27 downregulates Th2 cytokines (IL-4, IL-5), consistent with Lu et al. [12]. Although IL-27 has been reported to promote Th1 responses and IFN-γ [13,14], we observed no significant change in IFN-γ protein, in line with Suzuki et al. [15] and Hirahara et al. [16], suggesting complex regulatory mechanisms. While Hirahara et al. [16] and Ouyang et al. [17] reported that IL-27 inhibits Th17 differentiation in vitro, we found no effect on IL-17A in our in vivo model, possibly due to differences between in vitro and in vivo microenvironments. Systemic cytokine levels were nearly undetectable by CBA, contrasting with Lucas et al. [18], likely due to different models (OVA vs. HDM) or detection methods (ELISA vs. CBA). At the transcriptional level, IL-27 promoted T-bet and suppressed GATA-3 and RORγt mRNA, consistent with Lucas et al. [18] and Du et al. [19], indicating that IL-27 modulates Th1/Th2/Th17 imbalance at the transcriptional level. Interestingly, the 5.5-fold increase in T-bet mRNA did not translate to IFN-γ protein, suggesting that IL-27’s promotion of Th1 differentiation may not be entirely dependent on the classical IFN-γ pathway or may require longer action time [20]. Thus, in this model, IL-27 may restore immune balance mainly by inhibiting Th2/Th17 rather than strongly activating Th1, which may explain the controversial literature on IL-27 and Th1.

We observed that the proportions of *Lag3^+^* Tregs, CTLA4^+^ Tregs, and CD39^+^ Tregs were all significantly decreased in asthmatic mice, and IL-27 reversed these decreases (Figure 2B–D). *Lag3* transmits negative signals via MHC-II and enhances Treg suppression [21]. CTLA4 is a core contact-dependent suppressor [22,23], and CD39 hydrolyses ATP to adenosine. Their simultaneous downregulation on asthmatic Tregs may represent a molecular basis for impaired Treg function, and IL-27 restores their expression, suggesting it enhances Treg suppressive capacity via multiple pathways. Although some studies reported increased CD39 in asthmatic airways [24], our data on Treg-specific CD39 are consistent with reports that CD39^+^ Tregs control allergic responses [25].

In *Lag3*^−/−^ mice, IL-27 failed to mitigate inflammation, modulate Th responses, or increase Treg numbers, confirming that *Lag3* is essential for IL-27-mediated Treg regulation, corroborated by Nguyen et al. [26], who showed that *Lag3*-deficient Tregs cannot suppress inflammation upon IL-27 treatment. Although IL-27 increased CTLA4^+^ Tregs in WT, the low Treg frequency in *Lag3^−/−^* mice precluded further analysis, suggesting that *Lag3* is the primary target.

While the IL-27/*Lag3* axis in Tregs has been previously described by Do et al. [8] and Nguyen et al. [26], our study provides several distinct advances. First, we demonstrate this axis specifically in an HDM-induced asthma model, whereas previous work used OVA-based systems or adoptive transfer models. Second, we provide genetic evidence that *Lag3* is absolutely required for IL-27’s anti-inflammatory effects, as IL-27 completely failed to protect *Lag3^−/−^* mice, a finding not directly shown in prior reports. Third, we show that IL-27 simultaneously upregulates not only *Lag3* but also CTLA4 and CD39 on Tregs, suggesting a broader regulatory program. Thus, our work extends previous mechanistic insights to a more clinically relevant asthma model and identifies the IL-27/*Lag3* axis as a potential therapeutic target in allergic airway disease. Recent studies have further highlighted the role of Treg-expressed immune checkpoints in allergic inflammation [27,28,29,30], and our findings add that the IL-27/*Lag3* axis is a critical upstream regulator.

This study has several limitations. First, we used a preventive protocol (IL-27 administered 30 min before each challenge), which differs from the clinical scenario where asthma patients typically receive treatment after symptom onset; therefore, the therapeutic efficacy of IL-27 in established asthma remains to be determined. Second, we did not perform direct Treg functional assays, such as in vitro suppression assays, adoptive transfer experiments, or Treg depletion studies; our inference regarding Treg function is based on the upregulation of surface inhibitory molecules (*Lag3*, CTLA4, and CD39). Third, we did not evaluate proliferation markers (e.g., Ki67, BrdU incorporation) or apoptosis markers (e.g., Annexin V), so we cannot distinguish whether the increased Treg frequency results from enhanced proliferation, reduced apoptosis, or both. Fourth, the cellular source of IL-27 and whether it acts directly on Tregs via the IL-27 receptor remain unclear; these questions require Treg-specific IL-27 receptor conditional knockout mice for further clarification. Fifth, airway hyperreactivity was assessed in this study; however, the data are not presented in the current manuscript and will be reported separately in a future study. Sixth, we used only an acute asthma model; chronic and steroid-resistant models should be evaluated in future investigations to better reflect the heterogeneity of human asthma. Despite these limitations, our study provides a mechanistic basis for targeting the IL-27/*Lag3* axis in Treg-mediated therapy for allergic asthma.

## 4. Materials and Methods

### 4.1. Mice

Female wild-type (*WT*) C57BL/6 mice and *Lag3*- knockout (*Lag3*^−/−^) C57BL/6 mice, aged 6–8 weeks and weighing 18–22 g, were used in this study. *WT* mice were purchased from the Hubei Provincial Experimental Animal Research Center, and *Lag3*^−/−^ mice were obtained from GemPharmatech Co., Ltd. (Nanjing, China). All animals were housed under specific pathogen-free (SPF) conditions at the Animal Experiment Center of Wuhan University, with an ambient temperature of 22 ± 2 °C, relative humidity of 50 ± 10%, and a 12-h light/dark cycle. The mice had free access to autoclaved SPF commercial feed and acidified drinking water (pH 2.5–3.0). Animals were housed at a density of no more than five per cage (cage dimensions: 325 × 210 × 180 mm) with autoclaved corncob bedding, which was changed weekly. Environmental enrichment consisted of sterile cotton nestlets and a red polycarbonate tunnel toy (approximately 10 cm in length) in each cage; these were replaced or disinfected every two weeks. For experimental interventions, mice were first anesthetized with an intraperitoneal injection of sodium pentobarbital (50 mg/kg) and then administered house dust mite (HDM) solution (20 μL per mouse) via the intranasal route (n = 5). This procedure did not cause pain; therefore, no analgesic drugs were administered. The measured outcomes included daily general behavioral observations (fur condition, activity level, food and water intake, hunched posture, etc.), body weight changes recorded every three days (to the nearest 0.1 g), and survival rate. For tissue sampling, mice were euthanized by cervical dislocation without additional anesthesia, and samples were collected immediately after death. Euthanasia was performed by cervical dislocation either at the scheduled endpoint or when pre-established humane criteria were met. The criteria for early euthanasia were: body weight loss exceeding 20% of initial weight, absence of spontaneous activity and complete anorexia for more than three consecutive days, severe hunched posture with ruffled fur indicating a moribund state, or the development of severe infection or neurological symptoms (e.g., convulsions, paralysis). All experimental procedures were approved by the Animal Ethics Committee of Wuhan University (Ethics Approval No. ZN2022152).

### 4.2. HDM-Induced Acute Allergic Asthma Model

A stock solution of house dust mite (HDM) (Cat#XPB91D3A2.5; Stallergenes Greer, Greenville, NC, USA) extract was prepared at 1 µg/µL in phosphate-buffered saline (PBS) and stored at −20 °C. The sensitization solution (10 µL HDM + 20 µL PBS) and challenge solution (15 µL HDM + 15 µL PBS) were freshly prepared before each administration. Recombinant IL-27(Cat#abs04590; Absin Bioscience Inc., Shanghai, China) protein was diluted to 100 ng/µL in PBS and stored at −20 °C.

On day 1, mice were sensitized via intranasal instillation of the HDM sensitization solution under anesthesia induced by intraperitoneal injection of 1% sodium pentobarbital (50 mg/kg). From days 6 to 10, mice were challenged daily with the HDM challenge solution. On day 14, sample collection was performed. Thirty minutes before each challenge, mice in the treatment group received intranasal administration of 2 µg recombinant IL-27 in 20 µL PBS. Control mice received an equal volume of PBS at both sensitization and challenge time points (Figure 1A). Airway hyperreactivity was assessed; however, the data are not included in the present study.

### 4.3. Serum and Bronchoalveolar Lavage Fluid (BALF) Collection

Blood samples were collected from the orbital plexus using a heparinized capillary tube and transferred to centrifuge tubes. After blood collection, mice were euthanized by cervical dislocation. Blood samples were kept at 4 °C overnight to allow for serum separation and then centrifuged at 2000× *g* for 10 min at 4 °C (Beckman Coulter Avanti J-20XP centrifuge (Beckman Coulter, Inc., Brea, CA, USA). The supernatant (serum) was aliquoted and stored at −80 °C for further analysis.

Following euthanasia, the trachea was exposed and cannulated with a 24-G intravenous catheter. Lungs were lavaged three times with 0.5 mL of sterile PBS. BALF was collected, and only samples with a recovery rate ≥ 80% were used for subsequent analysis.

### 4.4. Flow Cytometry

Lung tissues were harvested and minced in 1 mg/mL collagenase type I solution (each lung was digested in a mixture of 1 mL of collagenase type I solution and 2 mL of PBS). After incubation at 37 °C for 1 h, the digested tissue was filtered through a 200-μm mesh and centrifuged at 380× *g* for 5 min. Erythrocytes were lysed, and the remaining cells were washed and resuspended in PBS. Cell concentration was adjusted to 1 × 10^6^ cells per tube for staining. Cells were stained with the following fluorochrome-conjugated antibodies at a 1:200 dilution: CD4-BV421 (Cat#100437, BioLegend, San Diego, CA, USA), Lag3-APC750 (Cat#125239, BioLegend, San Diego, CA, USA), CTLA4-APC750 (Cat#369627, BioLegend, San Diego, CA, USA), and CD39-PE (Cat#143803, BioLegend, San Diego, CA, USA). Surface staining was performed for 40 min at 4 °C in the dark. Cells were then fixed and permeabilized using a Foxp3 staining buffer set, followed by intracellular staining with Foxp3-Alexa Fluor^®^ 647 (Cat#126407, BioLegend, San Diego, CA, USA) at a 1:200 dilution for 50 min at room temperature. Finally, cells were washed and analyzed by flow cytometry(FACSAria™ III cell sorter (BD Biosciences, San Jose, CA, USA).

### 4.5. Enzyme-Linked Immunosorbent Assay (ELISA)

Levels of IL-4 (Cat#abs520003-96T), IL-5 (Cat#abs552005-96T), IL-10 (Cat#abs520005-96T), IL-17A (Cat#abs552807-96T), and IFN-γ (Cat#abs520007-96T) in BALF supernatants were measured using commercial ELISA kits (Absin, Shanghai, China) according to the manufacturer’s instructions.

### 4.6. Cytometric Bead Array (CBA)

Serum levels of IL-4, IL-5, IL-10, IL-17A, and IFN-γ were quantified using a CBA mouse inflammation kit (Cat#BK-CBA-4-01245-96T, Absin, Shanghai, China) following the manufacturer’s protocol.

### 4.7. Reverse Transcription Quantitative PCR (RT-qPCR)

Total RNA was extracted from lung tissues using Trizol reagent, followed by phase separation with chloroform, precipitation with isopropanol, and washing with ethanol. RNA purity and concentration were determined by spectrophotometry (A260/A280 ratio between 1.8 and 2.0). Gene-specific primers for T-bet, GATA-3, ROR-γT, and the reference gene GAPDH were designed using Primer 5.0 software (Table 1). qPCR was performed using SYBR Green master mix in a 25 µL reaction volume. Amplification was carried out using a two-step protocol on a real-time PCR system. Melting curve analysis was used to confirm amplification specificity. Relative gene expression was calculated using the 2^−ΔΔCt^ method with GAPDH as the internal control.

### 4.8. Histopathological Evaluation

#### 4.8.1. H&E Staining

Six complete small airway cross-sections per sample (200× magnification) were evaluated and scored according to the criteria listed in Table 2.

#### 4.8.2. PAS Staining

Six small airway cross-sections per sample were analyzed. The periodic acid–Schiff (PAS)-positive area (APSA) and the basement membrane perimeter (Pbm) were measured using CaseViewer software (version number2.4.0.119028). The APSA/Pbm ratio was calculated to quantify goblet cell hyperplasia.

### 4.9. Statistical Analysis

All statistical analyses were performed using GraphPad Prism 10. For comparisons involving more than two groups, one-way ANOVA followed by Tukey’s post hoc test was used. For direct comparisons between two groups, unpaired Student’s *t*-test was applied where appropriate. Data are presented as mean ± SD. A *p*-value < 0.05 was considered statistically significant.

## 5. Conclusions

The anti-asthmatic effect of exogenous IL-27 is associated with increased Treg frequency and upregulation of inhibitory markers, with *Lag3* serving as a pivotal target on Tregs.

## Figures and Tables

**Table 1:** qPCR Primer Sequence Information.

Gene	Primer Sequence		Size
*T-bet*	F	5′-GGTGTCTGGGAAGCTGAGAG-3′	122 bp
	R	5′-TGAAGGACAGGAATGGGAAC-3′	
*GATA-3*	F	5′-AGGGACATCCTGCGCGAACTGT-3′	166 bp
	R	5′-CATCTTCCGGTTTCGGGTCTGG-3′	
*ROR-γt*	F	5′-GCGGCTTTCAGGCTTCATGGAG-3′	221 bp
	R	5′-GGGCGCTGAGGAAGTGGGAAAA-3′	
*GAPDH*	F	5′-TGTGTCCGTCGTGGATCTGA-3′	150 bp
	R	5′-TTGCTGTTGAAGTCGCAGGAG-3′	

Note: F stands for Forward, R for Reverse.

**Table 2:** Scoring Criteria for Hematoxylin and Eosin (H&E) Staining.

Score	Histopathological Criteria
0	Intact lung architecture with no evident inflammatory cell infiltration around the bronchi and blood vessels or in the interstitium.
1	Essentially intact lung architecture with scattered inflammatory cells around the bronchi and blood vessels.
2	Mild destruction of lung architecture; focal aggregates of inflammatory cells (approximately 1–5 layers thick) are observed around the bronchi and blood vessels.
3	Moderate to severe destruction of lung architecture; dense peribronchial and perivascular inflammatory cell cuffs (>5 layers thick) are present, accompanied by extensive inflammatory cell infiltration in the pulmonary interstitium.

## Data Availability

The original data for this article are available from: https://doi.org/10.6084/m9.figshare.32330331.

## Abbreviations

The following abbreviations are used in this manuscript:

BALF	Bronchoalveolar lavage fluid
CBA	Cytometric bead array
ELISA	Enzyme-Linked Immunosorbent Assay
HDM	House dust mite
*Lag3*	*Lymphocyte-activation gene 3*
OVA	Ovalbumin
PAS	Periodic acid–Schiff
PBS	Phosphate-buffered saline
Tregs	Regulatory T cells
*WT*	*Wild-type*

## References

[1] Hammad H., Lambrecht B.N. (2021). The basic immunology of asthma. Cell.

[2] Dikiy S., Rudensky A.Y. (2023). Principles of regulatory T cell function. Immunity.

[3] Jiang H., Wu X., Zhu H., Xie Y., Tang S., Jiang Y. (2015). Foxp3^+^ Treg/Th17 cell imbalance in lung tissues of mice with asthma. Int. J. Clin. Exp. Med..

[4] Cui X., Liu W., Jiang H., Zhao Q., Hu Y., Tang X., Liu X., Dai H., Rui H., Liu B. (2024). IL-12 family cytokines and autoimmune diseases: A potential therapeutic target?. J. Transl. Autoimmun..

[5] Lu D., Lu J., Ji X., Ji Y., Zhang Z., Peng H., Sun F., Zhang C. (2020). IL-27 suppresses airway inflammation, hyperresponsiveness and remodeling via the STAT1 and STAT3 pathways in mice with allergic asthma. Int. J. Mol. Med..

[6] Chen X., Deng R., Chi W., Hua X., Lu F., Bian F., Gao N., Li Z., Pflugfelder S.C., de Paiva C.S. (2019). IL-27 signaling deficiency develops Th17-enhanced Th2-dominant inflammation in murine allergic conjunctivitis model. Allergy.

[7] Wang J., Klein C., Cochran J.R., Sockolosky J., Lippow S.M. (2025). Exploring new frontiers in LAG-3 biology and therapeutics. Trends Pharmacol. Sci..

[8] Do J., Visperas A., Sanogo Y.O., Bechtel J.J., Dvorina N., Kim S., Jang E., Stohlman S.A., Shen B., Fairchild R.L. (2016). An IL-27/Lag3 axis enhances Foxp3^+^ regulatory T cell-suppressive function and therapeutic efficacy. Mucosal Immunol..

[9] Schiffer M., Peters K., Peters M. (2022). Comparison of Airway Remodeling in Two Different Endotypes of Allergic Asthma. Int. Arch. Allergy Immunol..

[10] Yasuda Y., Nagano T., Kobayashi K., Nishimura Y. (2020). Group 2 Innate Lymphoid Cells and the House Dust Mite-Induced Asthma Mouse Model. Cells.

[11] Li X., Zhou L., Zhang Z., Liu Y., Liu J., Zhang C. (2020). IL-27 alleviates airway remodeling in a mouse model of asthma via PI3K/Akt pathway. Exp. Lung Res..

[12] Lu J., Ji X., Wang L., Sun F., Huang C., Peng H., Jiang Y., Guo Z., Liu X., Ji Y. (2022). Interleukin-27 ameliorates allergic asthma by alleviating the lung Th2 inflammatory environment. Int. J. Mol. Med..

[13] Xiong P., Liu T., Huang H., Yuan Y., Zhang W., Fu L., Chen Y. (2022). IL-27 overexpression alleviates inflammatory response in allergic asthma by inhibiting Th9 differentiation and regulating Th1/Th2 balance. Immunopharmacol. Immunotoxicol..

[14] Yoshida H., Nakaya M., Miyazaki Y. (2009). Interleukin 27: A double-edged sword for offense and defense. J. Leukoc. Biol..

[15] Suzuki M., Yokota M., Ozaki S., Matsumoto T. (2019). Intranasal Administration of IL-27 Ameliorates Nasal Allergic Responses and Symptoms. Int. Arch. Allergy Immunol..

[16] Hirahara K., Ghoreschi K., Yang X., Takahashi H., Laurence A., Vahedi G., Sciumè G., Hall A.O., Dupont C.D., Francisco L.M. (2012). Interleukin-27 priming of T cells controls IL-17 production in trans via induction of the ligand PD-L1. Immunity.

[17] Ouyang H., Cheng J., Du J., Gan H., Zheng L. (2020). Interleukin-27 Suppresses T Helper-17 Inflammation in Allergic Rhinitis. Iran. J. Immunol. IJI.

[18] Lucas S., Ghilardi N., Li J., de Sauvage F.J. (2003). IL-27 regulates IL-12 responsiveness of naive CD4^+^ T cells through Stat1-dependent and -independent mechanisms. Proc. Natl. Acad. Sci. USA.

[19] Du H., Wang Q., Ji J., Shen B., Wei S., Liu L., Ding J., Ma D., Wang W., Peng J. (2013). Expression of IL-27, Th1 and Th17 in patients with aplastic anemia. J. Clin. Immunol..

[20] Kamiya S., Owaki T., Morishima N., Fukai F., Mizuguchi J., Yoshimoto T. (2004). An indispensable role for STAT1 in IL-27-induced T-bet expression but not proliferation of naive CD4^+^ T cells. J. Immunol..

[21] Huang C., Workman C.J., Flies D., Pan X., Marson A.L., Zhou G., Hipkiss E.L., Ravi S., Kowalski J., Levitsky H.I. (2004). Role of LAG-3 in regulatory T cells. Immunity.

[22] Schmidt A., Oberle N., Krammer P.H. (2012). Molecular mechanisms of treg-mediated T cell suppression. Front. Immunol..

[23] Kawayama T., Matsunaga K., Kaku Y., Yamaguchi K., Kinoshita T., O’Byrne P.M., Hoshino T. (2013). Decreased CTLA4^+^ and Foxp3^+^ CD25^high^CD4^+^ cells in induced sputum from patients with mild atopic asthma. Allergol. Int. Off. J. Jpn. Soc. Allergol..

[24] Li P., Gao Y., Cao J., Wang W., Chen Y., Zhang G., Robson S.C., Wu Y., Yang J. (2015). CD39^+^ regulatory T cells attenuate allergic airway inflammation. Clin. Exp. Allergy J. Br. Soc. Allergy Clin. Immunol..

[25] Lynch J.P., Werder R.B., Curren B.F., Sikder M.A.A., Ullah A., Sebina I., Rashid R.B., Zhang V., Upham J.W., Hill G.R. (2020). Long-lived regulatory T cells generated during severe bronchiolitis in infancy influence later progression to asthma. Mucosal Immunol..

[26] Nguyen Q.T., Jang E., Le H.T., Kim S., Kim D., Dvorina N., Aronica M.A., Baldwin W.M.R., Asosingh K., Comhair S. (2019). IL-27 targets Foxp3^+^ Tregs to mediate antiinflammatory functions during experimental allergic airway inflammation. Jci Insight.

[27] Kim D., Kim G., Yu R., Lee J., Kim S., Qiu K., Montauti E., Fang D., Chandel N.S., Choi J. (2023). Lymphocyte activation gene 3 (Lag3) supports Foxp3^+^ Treg cell function by restraining c-Myc-dependent aerobic glycolysis. bioRxiv Prepr. Serv. Biol..

[28] Saheb Sharif-Askari F., Zakri A.M., Alenazy M.F., El-Wetidy M.S., Khalid Salah Al-Sheakly B., Saheb Sharif-Askari N., ALKufeidy R.M., Omair M.A., Al-Muhsen S., Halwani R. (2024). IL-35 promotes IL-35^+^IL-10^+^ Bregs and Conventional LAG3^+^ Tregs in the lung tissue of OVA-Induced Asthmatic Mice. Inflamm. Res..

[29] Martín-Cruz L., Benito-Villalvilla C., Sirvent S., Angelina A., Palomares O. (2024). The Role of Regulatory T Cells in Allergic Diseases: Collegium Internationale Allergologicum (CIA) Update 2024. Int. Arch. Allergy Imm.

[30] Kim D., Kim G., Yu R., Lee J., Kim S., Gleason M.R., Qiu K., Montauti E., Wang L.L., Fang D. (2024). Inhibitory co-receptor Lag3 supports Foxp3^+^ regulatory T cell function by restraining Myc-dependent metabolic programming. Immunity.

